# Perceptions of intensive care unit health care professionals in Brazil regarding postintensive care syndrome: a survey study

**DOI:** 10.62675/2965-2774.20250407

**Published:** 2025-12-08

**Authors:** José Mário Meira Teles, Fernanda Saboya R. Almendra, João Gabriel Rosa Ramos, Zilfran Teixeira Carneiro, Marcelle Passarinho Maia, Lucio Couto de Oliveira, Gabriela Soares Rech, Duane Mocellin, Regis Goulart Rosa, Rodrigo Meira-Teles, Cassiano Teixeira

**Affiliations:** 1 Hospital Municipal de Salvador Intensive Care Unit Salvador BA Brazil Intensive Care Unit, Hospital Municipal de Salvador - Salvador (BA), Brazil.; 2 Hospitais Rede D'Or Regional Zona Sul Rio de Janeiro RJ Brazil Hospitais Rede D'Or Regional Zona Sul - Rio de Janeiro (RJ), Brazil.; 3 Clínica Florence Salvador BA Brazil Clínica Florence - Salvador (BA), Brazil.; 4 Hospital Universitário Walter Cantideo Fortaleza CE Brazil Hospital Universitário Walter Cantideo - Fortaleza (CE), Brazil.; 5 Hospital Santa Lúcia Sul e Norte Brasília DF Brazil Hospital Santa Lúcia Sul e Norte - Brasília (DF), Brazil.; 6 Hospital Geral Clériston Andrade Feira de Santana BA Brazil Hospital Geral Clériston Andrade - Feira de Santana (BA), Brazil.; 7 Hospital Moinhos de Vento Porto Alegre RS Brazil Hospital Moinhos de Vento - Porto Alegre (RS), Brazil.; 8 Universidade Federal do Rio Grande do Sul Intensive Care Department Porto Alegre RS Brazil Intensive Care Department, Universidade Federal do Rio Grande do Sul - Porto Alegre (RS), Brazil.; 9 Faculdade de Medicina ZARNS Salvador BA Brazil Faculdade de Medicina ZARNS - Salvador (BA), Brazil.; 10 Universidade Federal de Ciências da Saúde de Porto Alegre Internal Medicine and Rehabilitation Sciences Department Porto Alegre RS Brazil Internal Medicine and Rehabilitation Sciences Department, Universidade Federal de Ciências da Saúde de Porto Alegre - Porto Alegre (RS), Brazil.

**Keywords:** Critical care, Health personnel, Awareness, Quality of life, Surveys and questionnaires

## Abstract

**Objective:**

To assess the perceptions of intensive care unit health care professionals in Brazil regarding postintensive care syndrome and the importance attributed to it by individuals and institutions.

**Methods:**

A web-based survey was conducted among intensive care unit professionals across all five Brazilian geopolitical regions. The questionnaire was used to collect demographic and professional data and to explore participants’ perceptions of postintensive care syndrome, including a focus on patient/family-centered outcomes and long-term intensive care unit consequences.

**Results:**

A total of 1,527 intensive care unit professionals responded, 61.3% of whom were women. The responses represented 12 professional categories, including physicians (51.1%), physiotherapists (16.9%), nurses (12.7%), and psychologists (5.8%). Among the participants, 50.4% had training or certification in critical care, and 59.9% had more than five years of experience. However, 24% had never heard of postintensive care syndrome. Awareness was significantly higher among those with specialized training (85.2% *versus* 66.6%; p < 0.001). Only 26.4% reported that their institutions had protocols for postintensive care syndrome assessment before hospital discharge. A significant difference emerged between individual and institutional priorities regarding patient/family-centered outcomes and postintensive care unit care (p < 0.001). In 60% of the cases, intensive care unit teams were not involved in patients’ hospital discharge.

**Conclusion:**

Despite moderate awareness of postintensive care syndrome among intensive care unit professionals, there is a considerable gap between staff and the institutional prioritization of postintensive care unit care in Brazil. This highlights the need to increase awareness and develop structured postintensive care unit care protocols, ensuring improved long-term outcomes for intensive care unit patients and their families.

## INTRODUCTION

Advancements in critical care have led to a growing population of intensive care unit (ICU) survivors. The long-term impact of critical illness on both ICU survivors and their families is increasingly recognized and often persists for months or even years beyond hospitalization.^(1,2)^ Postintensive care syndrome (PICS) is characterized by new or worsening physical, cognitive, or mental health impairments that endure after an acute hospital stay.^(1,2)^ Its impact extends beyond patients, affecting their loved ones as well; this phenomenon is referred to as postintensive care syndrome–family (PICS-F).^(1,3)^ Studies estimate that between 50% and 100% of ICU survivors experience at least one physical symptom or impairment linked to PICS, such as weakness, pain, fatigue or the development of new or worsened functional impairments.^(1–3)^ The prevalence of psychological disorders—including depression, anxiety, and posttraumatic stress disorder—ranges from 8% to 80%, with considerable overlap among these conditions.^(4,5)^ Cognitive deficits following an ICU stay are also common, with reported prevalence varying from 10% to 100%.^(2,6,7)^ The aftermath of critical illness can permeate nearly every facet of life, leaving both patients and families to navigate significant challenges. Beyond coping with these burdens, many patients and families also struggle with inadequate postdischarge support, often finding the health care system difficult to navigate.^(1–3,8–12)^

Given these far-reaching consequences, preventing, identifying, and managing PICS and PICS-F are crucial to optimizing recovery and helping patients reclaim their lives.^(9,13)^ However, achieving this requires ICU professionals to recognize their signs and symptoms.^(14,15)^ Despite the importance of post-ICU care, research on health care providers’ perspectives regarding long-term outcomes in Brazil remains scarce. While some awareness of PICS and PICS-F exists among Brazilian ICU professionals, substantial gaps persist in both knowledge and practice, particularly beyond the ICU environment. The aim of this study is to assess how familiar ICU health care providers in Brazil are with PICS and PICS-F and to evaluate the importance they and their institutions assign to these conditions.

Therefore, the goal of this study was to assess the knowledge of ICU health care professionals in Brazil regarding PICS and PICS-F and the importance attributed to these conditions by individuals and institutions.

## METHODS

### Design

The survey was developed and administered by the Post-ICU Care Committee of the *Associação de Medicina Intensiva Brasileira* (AMIB) to support the management of educational processes using a convenience sample of health care professionals working in ICUs across five regions of Brazil. The Post-ICU Care Committee is composed of intensive care specialists with at least 10 years of professional practice, publication of scientific papers or participation in research projects related to PICS, and extensive experience in the subject being researched. The group comprised 11 specialists, including 7 physicians, 2 psychologists, 1 nurse and 1 physiotherapist.

The study questionnaire was reviewed by the Research Ethics Committee of *Hospital Moinhos de Vento*, which waived the need for formal evaluation in accordance with Resolution 510/2016 of the National Health Council, as it qualifies as public opinion research with unidentified participants. Prior to completing the questionnaire, the participants were informed about the objectives of the study, the importance of anonymity and the voluntary nature of their participation. Given that the research did not involve the collection of personal information, it was not necessary to use a formal consent form. Participants did not receive financial compensation, and the data collected were stored by four principal investigators, without a confidentiality agreement between them.

### Development of the questionnaire

To ensure the questionnaire's relevance and comprehensiveness, the instrument's construction followed a structured approach, initially combining a nonsystematic review of the PubMed literature and seeking to identify the main aspects related to PICS that should be addressed in the survey. Furthermore, a comprehensive analysis of the remaining studies on the subject, along with analogous research conducted internationally, was undertaken to adapt methodologies that had already been validated in the Brazilian context.

In this study, a process of gathering and refining expert opinion to reach consensus in iterative rounds was used; this method was similar to the Delphi method but without anonymity and without the use of quantitative Likert-type scales for responses. After four iterations, the fifth version of the questionnaire was approved, containing a total of 63 questions, eight of which were adaptive, allowing participants to skip certain questions on the basis of their previous answers. A pilot study was then conducted with 10 health care professionals working in the ICU of the *Hospital Municipal de Salvador* (HMS). The aim of this study was to evaluate the functionality of the final version of the questionnaire to ensure that the questions were understood and to evaluate the average response time, leading to modifications in the questionnaire.

The final survey was structured into four main sections, covering the following: 1. Sociodemographic and professional data, 2. Perceptions of PICS/PICS-F, 3. Institutional practices in the prevention of PICS as perceived by the participants, and 4. ICU postdischarge care plan (Chart 1S - Supplementary Material).

In addition, the questionnaire included a Likert-type scale to measure the participants’ perceptions of the importance and level of agreement of each outcome for the patient's quality of life on a scale of 1 (not important) to 5 (very important). Similarly, questions employing the Likert scale were incorporated to assess the participants’ perceptions of the institution's prioritization of the prevention of PICS and its outcomes on a scale from 1 (not a priority) to 5 (high priority).

The survey design followed the CHERRIES checklist methodological guidelines,^(16)^ ensuring transparency in data collection and in the reporting of the results.

### Administration of the questionnaire

Participants were eligible if they were clinicians who worked in the intensive care unit or with critically ill patients. There were no restrictions regarding professional category or specific training in intensive care.

The data collection period spanned from November 10 to December 10, 2022. Participants were initially recruited during the Brazilian Congress of Intensive Care Medicine (CBMI), held between November 10 and 12, 2022, with approximately 3,000 participants, where AMIB representatives promoted the survey in person at the congress stand and through the event's official app. Subsequent to the congress, from November 13 to December 10, 2022, invitations were extended through various social media networks, the CBMI mobile application and the official website of the AMIB.

The survey was administered via a web link through Google Forms and was used exclusively for data collection. The sequence of the questions was not randomized. The survey format was designed to accommodate up to eight printed pages or 11 screens in the digital environment. Participants were permitted to return to previously completed questions without the requirement of confirming their answers prior to advancing to subsequent questions.

The survey completion rate could not be calculated, as the system used did not record the number of unique visitors. Consequently, it was not feasible to ascertain the participation rate or the completion rate, as the system did not permit tracking the number of people who accessed the questionnaire or differentiating between participants who only viewed and those who actually answered.

To mitigate the occurrence of duplicate responses, participants were explicitly instructed to refrain from submitting multiple answers. Although it was possible to identify duplicated responses based on similar date/time stamps and birth year, which could eliminate response errors, technical methods such as the use of cookies, IP restriction, unique tokens, or login authentication were not employed, as the implementation of such systems has been shown to reduce response rates. The credibility of the AMIB and the ethical commitment of the participants were sufficient to guarantee the authenticity of the responses. The nature of the questionnaire, devoid of any significant disruption to the participants’ usual practices, further reduced the probability of intentional multiple responses.

Responses to all the questions were mandatory, with the exception of the adaptive questions. If a participant inadvertently exited the survey, the system enabled them to resume filling it out without the answers they had previously recorded being forfeited. No exclusions were made on the basis of the time taken to complete the questionnaire. However, four questionnaires were excluded because they were completed outside of Brazil. The full design and implementation of the survey process are depicted in the flowchart presented in figure 1S (Supplementary Material).

### Statistical analysis

Descriptive statistical analysis was applied as appropriate, with categorical variables presented as absolute and relative frequencies and continuous variables expressed as median values with interquartile ranges (IQRs) to summarize the data for nonnormally distributed variables. The Shapiro–Wilk test was used to assess the normality of the distribution.

Likert scales were categorized into bivariate dummy variables to allow for further analyses. Responses from 1 to 3 were categorized as not important/not priority, and responses of 4 and 5 were categorized as important/priority.

The chi-square test was employed to assess differences in proportions, while the Mann–Whitney U test was utilized to assess differences in ranks. The p value threshold for statistical significance was set at < 0.05. All analysis were conducted using the R Statistical Package (Version 4.0.3) and Microsoft Excel.

## RESULTS

A total of 1,527 Brazilian health care professionals responded to the survey, and 61.3% (n= 936) of the respondents were women. Among the participants, 51.1% were physicians, 50.4% were board certificated or board eligible in intensive care unit care (i.e., completed a medical or multiprofessional residency in intensive care or were board certified by AMIB), and 59.9% had more than five years of experience. The responses were representative of all five geopolitical regions of Brazil and encompassed a diverse range of professional categories. The main demographic characteristics of the respondents are presented in table 1.

**Table 1:** Demographics of the survey responders

Characteristics		Value
Age		38 (33; 46) (1,519)
Sex		
	Female	936/15,27 (61.3)
Profession		
	Physician	780/1,527 (51.1)
	Physiotherapist	258/1,527 (16.9)
	Nurse	194/1,527 (12.7)
	Psychologist	88/1,527 (5.8)
	Nutritionist	76/1,527 (5.0)
	Speech therapist	65/1,527 (4.3)
	Pharmacist	27/1,527 (1.8)
	Dentist	13/1,527 (0.9)
	Occupational therapist	10/1,527 (0.7)
	Nursing technician	8/1,527 (0.5)
	Social worker	7/1,527 (0.5)
	Music therapist	1/1,527 (0.1)
	Board certificated or board eligible in intensive care	769/1,527 (50.4)
Experience in intensive care (years)		
	0 - 5	612/1,527 (40.1)
	6 - 10	338/1,527 (22.1)
	11 - 15	230/1,527 (15.1)
	16 - 20	138/1,527 (9.0)
	More than 20	209/1,527 (13.7)
Region of the country		
	North	91/1,525 (6.0)
	Northeast	523/1,525 (34.3)
	Central–West	204/1,525 (13.4)
	Southeast	491/1,525 (32.2)
	South	216/1,525 (14.2)

Data are expressed as medians (interquartile ranges) and n (%).

### Perceptions regarding postintensive care syndrome

A total of 24.1% (n= 367) of the respondents reported having never heard the term PICS before. Awareness of the syndrome was notably higher among those with specialized training or certification (85.2% *versus* 66.6%; p < 0.001) and among physicians in comparison to other healthcare categories (54.2% *versus* 45.8%, p < 0.001).

### Practices and postdischarge care plan

Only 26.4% of the health care institutions represented by the respondents have established formal protocols for assessing patients’ needs and determining the appropriate level of care required following their discharge from the hospital. Moreover, in 60% of the cases, ICU teams were not directly involved in the hospital discharge process. For those 40% who participate in the hospital discharge process, the responsibility for this transition is frequently borne by physicians (84.2%), nurses (75.5%), or physiotherapists (72.1%), with minimal involvement from other health care professionals (Table 1S - Supplementary Material).

There was great variation regarding strategies implemented to prevent PICS. Only 1,017 (66.4%) of the respondents reported the existence of an analgesia protocol, 682 (44.5%) reported the systematic application of pain assessment tools, and 614 (40.1%) reported the administration of preemptive analgesia. In addition, 931 (60.8%) reported a sedation protocol, with 703 (45.9%) reporting the systematic application of sedation assessment tools, 572 (37.4%) the systematic application of delirium screening tools and 537 (35.1%) the systematic application of nonpharmacological measures to prevent *delirium*. Moreover, 1,137 (74.3%) reported the promotion of early mobilization, 450 (29.4%) the promotion of patient sleep quality, 1,078 (70.4%) a flexible visit policy, 1,209 (79%) psychological support for patients and 1,067 (69.7%) psychological support for family members. The rates of screening before ICU discharge were low, with 225 (14.7%) reporting systematic screening for high-risk patients for PICS and 133 (8.7%) reporting systematic screening for high-risk family members for PICS-F. Furthermore, 338 (22.1%) reported offering a cognitive assessment before ICU discharge, 707 (46.2%) offered a motor assessment before ICU discharge, and 408 (26.6%) offered a psychological assessment before ICU discharge.

A significant discrepancy was identified (p < 0.001) between the level of importance respondents assigned to each postintensive care outcome and the level they believed their institutions prioritized, with personal perceptions consistently being rated higher than institutional priorities. This discrepancy was particularly evident for patient-centered outcomes (Table 2) and family-centered outcomes (Table 3).

**Table 2:** Comparison between respondents’ and their institutions’ priority perceptions regarding postintensive care outcomes among patients

Outcome	Respondent priority	Institutional priority	p value
Unplanned ICU readmission	1,381 (90.4)	968 (63.3)	< 0.001
Anxiety	1,315 (86.1)	713 (46.6)	< 0.001
Depression	1,398 (91.5)	721 (47.2)	< 0.001
PTSD	1,407 (92.1)	705 (46.1)	< 0.001
Sleep disturbances	1,362 (89.1)	683 (44.7)	< 0.001
Cognitive dysfunction	1,374 (89.9)	769 (50.3)	< 0.001
Muscle weakness	1,408 (92.2)	984 (64.4)	< 0.001
Physical capacity	1,377 (90.1)	949 (62.1)	< 0.001
Chronic pain	1,365 (89.3)	907 (59.4)	< 0.001
Muscle contractures	1,204 (78.8)	767 (50.2)	< 0.001
Dysphagia	1,326 (86.8)	977 (63.9)	< 0.001
Malnutrition	1,362 (89.1)	1,069 (70.0)	< 0.001
Sexual dysfunction	1,006 (65.8)	426 (27.9)	< 0.001
Return to work	1,304 (85.4)	678 (44.4)	< 0.001
Financial Stress	1,287 (84.2)	587 (38.4)	< 0.001

ICU - intensive care unit, PTSD - posttraumatic stress disorder. Number of respondents = 1527. Data are expressed as n (%).

**Table 3:** Comparison between respondents’ and their institutions’ priority perceptions regarding postintensive care outcomes for family members

Outcome	Respondent priority	Institutional priority	p value
Anxiety	1,366 (89.4)	707 (46.3)	< 0.001
Depression	1,365 (89.3)	647 (42.3)	< 0.001
PTSD	1,343 (87.9)	610 (39.9)	< 0.001
Pathological grief	1,379 (90.3)	733 (48.0)	< 0.001
Financial stress	1,379 (90.3)	543 (35.5)	< 0.001

PTSD – posttraumatic stress disorder. Data are expressed as n (%).

## DISCUSSION

The main finding of this study is that most health care professionals are aware of the long-term effects on ICU survivors, such as physical, cognitive, and mental health issues. However, there is a significant lack of formal protocols in hospitals to address these impacts. While most respondents had heard of PICS and PICS-F, only a small proportion of the institutions had protocols for postdischarge care. Additionally, ICU teams were frequently not involved in the transition from the ICU to the ward, with only a few professionals actively participating.

Barriers to the implementation of adequate post-ICU care can be divided into three categories: lack of awareness among health care professionals, lack of institutional recognition, and the absence of standardized protocols for identifying and managing these patients.

With respect to the knowledge of health professionals regarding PICS and PICS-F, we found that only 26.4% of the respondents had never heard of PICS before, but most respondents did not have processes in place to prevent and manage this syndrome. In agreement, a recent study developed and validated a test (PICS-KT, knowledge test) among 117 doctors and nurses who had worked in ICUs for at least six months to assess ICU professionals’ awareness and understanding of the syndrome. The results revealed that most ICU professionals had a strong understanding of interventions for preventing and managing PICS, with the PICS-KT showing a high Cronbach α reliability coefficient of 0.93.^(14)^ In contrast, another study examined how doctors and nurses in medical and surgical wards in Denmark handled postintensive care impairments. While most participants reported having average or greater knowledge about the hospital needs of ICU patients, few conducted routine screening for physical, psychological, and cognitive impairments related to ICU stays, with screening rates of 22.9%, 70.7%, and 57.3%, respectively.^(15)^

There are few surveys^(1,2,13,14)^ regarding strategies implemented for the prevention and management of PICS. The variation in priorities between respondents and institutions suggests that further efforts may be necessary to align institutional policies with the evolving understanding of post-ICU recovery and the comprehensive needs of patients and their families.^(3)^ In a recent retrospective cohort study that included 847 patients transferred from an ICU or intermediate care unit to a postacute care facility between July 2017 and April 2023, 82% of admissions were for rehabilitation, and only 18% were for palliative care. Of these, 45.9% were discharged home, 20.4% returned to acute care hospitals, and 33.6% died in the hospital (92% with a do-not-resuscitate order). Overall, 63% improved their functional status, and 27.6% had no change.^(12)^ A multicenter prospective cohort study was conducted in 10 Brazilian tertiary hospital ICUs, analyzing 1,554 adult ICU survivors with prolonged hospitalizations, and the cumulative 12-month mortality was 28.2% (7.9% early mortality [30 days] and 22.3% late mortality [31 - 365 days]). Infections were the primary cause of mortality in both periods, accounting for 47.2% of early deaths and 36.4% of late deaths. The risk factors for early mortality included age over 65 years, advanced pre-ICU comorbidities, physical dependence prior to ICU admission, ICU-acquired infections, ICU readmissions, and severity of illness at the time of ICU admission. Similarly, late mortality was associated with comparable factors and was markedly linked to ICU readmission.^(16)^ It is recommended that modifiable and potentially modifiable risk factors be identified at the time of admission to the ICU and managed in accordance with the best available evidence from the literature to reduce the occurrence of not only PICS but also of other negative long-term outcomes.

This study was the first survey in Brazil regarding postintensive care syndrome and, to our knowledge, it is also the first conducted in Latin America, representing an addition to the literature in this field. This survey was able to encompass a large number of respondents from diverse backgrounds and raised several issues that may help formulate education and practice policies related to this topic. Nevertheless, our study has several limitations. First, it was a convenience sample (i.e., nonrandom), but it was a large survey, including multiple categories of intensive care clinicians from all Brazilian regions. Second, physicians were overrepresented in the sample, so these results may not be representative of all specialties involved in the care of critically ill patients, especially those that work more directly with some of the sequelae of PICS, such as physical therapists, occupational therapists and psychologists. Third, 40% of the participants had less than 5 years of experience; thus, the high degree of awareness of PICS may reflect recent practice changes and may not reflect overall awareness. Finally, processes and protocols were not directly assessed, as the survey assessed the perceptions and awareness of clinicians regarding the existence of such protocols and the priorities set by the institution. However, because the survey was designed to assess clinicians’ perceptions, these results are aligned with the purpose of the study and reflect the actual practices as perceived by the clinicians.

## CONCLUSION

In this survey of Brazilian intensivists, health care professionals recognize the potential long-term effects experienced by intensive care unit survivors. However, it is evident that the hospitals where they work lack established protocols to mitigate these impacts. These results may help clinicians, hospital administrators, and policy-makers evaluate strategies to mitigate the burden of postintensive care syndrome.

## Data Availability

Data is available on demand from referees.
