# Supplementary material for: Perceptions of intensive care unit health care professionals in Brazil regarding postintensive care syndrome: a survey study
Source: Crit Care Sci. 2025 Dec 8;37:e20250407. doi: 10.62675/2965-2774.20250407 (PMC12711214; doi:10.62675/2965-2774.20250407)
Supplement: Supplementary Material [file 2965-2774-ccsci-37-e20250407-Suppl01.pdf]

Critical Care Science
https://doi.org/10.62675/2965-2774.20250407

SUPPLEMENTARY MATERIAL

Perceptions of intensive care unit healthcare professionals in Brazil about post-intensive care syndrome: a survey study

José Mário Meira Teles[1], Fernanda Saboya R. Almendra[2], João Gabriel Rosa Ramos[3], Zilfran Teixeira Carneiro[4], Marcelle Passarinho Maia[5], Lucio Couto de Oliveira Junior[6], Gabriela Soares Rech[7], Duane Mocellin[7], Regis Goulart Rosa[8], Rodrigo Meira-Teles[9], Cassiano Teixeira[10]

CHART 1S - SURVEY

Evaluation of intensive care unit professionals' knowledge, perception of the importance, practice, and care plan of postintensive care unit syndrome

Since 2010, specialists in the care of critically ill patients have been working to identify and prevent a series of problems commonly experienced by intensive care unit (ICU) survivors. All of these problems, which affect the cognitive, physical, and psychological spheres and lead to a reduction in quality of life, have been called post-ICU syndrome (PICS).
The objective of this survey is to ascertain the conditions of care, knowledge, and practices adopted by members of intensive care teams regarding PICS.
Participation in this survey is voluntary, and those who elect to participate are asked to complete the survey. The survey is estimated to require approximately five minutes to complete. If you do not wish to participate, you may simply close the browser page. It is imperative to note that no personally identifiable information will be collected, and the integrity of the survey responses will be maintained.
Your participation is greatly appreciated, as your contributions play a pivotal role in advancing our understanding of this crucial subject.

Would you like to take part in a survey to determine the care conditions, knowledge and practices of intensive care team members regarding post-ICU syndrome?

- ◯ Yes
- ◯ No

1. Sociodemographic data

1.1. What year were you born? (Format: yyyy) _________

1.2. What is your sex?

- ◯ Female
- ◯ Male
- ◯ I prefer not to say

1.3. What is your professional category?

- ☐ Physician
- ☐ Registered nurse
- ☐ Physiotherapist

This is an open access article under the CC BY license (https://creativecommons.org/licenses/by/4.0/).

- ☐ Psychologist
- ☐ Nutritionist
- ☐ Speech therapist
- ☐ Dentist
- ☐ Pharmacist
- ☐ Social worker
- ☐ Other

1.4. Do you have an intensive care degree or residency?

- ○ Yes
- ○ No

1.5. Length of time working in critical care

- ○ 0 - 5 years
- ○ 6 - 10 years
- ○ 11 - 15 years
- ○ 16 - 20 years
- ○ More than 20 years

1.6. In which region of the country is the organisation you work for located?

- ○ North
- ○ North-East
- ○ Centre West
- ○ South-East
- ○ South
- ○ Other

2. Knowledge and importance

2.1. Have you heard of post-ICU syndrome (PICS)?

- ○ Yes
- ○ No

2.2. Evaluate the importance of each of the outcomes for the PATIENT'S Quality of Life

Consider your experience and professional opinion. Please indicate the option that is considered most appropriate on a scale of 1 (not important) to 5 (very important).

Unplanned readmission

1	2	3	4	5
○	○	○	○	○

Anxiety

1	2	3	4	5
○	○	○	○	○

Depression

1	2	3	4	5
○	○	○	○	○

Post-traumatic stress

1	2	3	4	5
○	○	○	○	○

Sleep disorder

1	2	3	4	5
○	○	○	○	○

Cognitive dysfunction

1	2	3	4	5
○	○	○	○	○

Independence for daily activities (hygiene, eating, walking, bed-chair transfer, etc.)

1	2	3	4	5
○	○	○	○	○

Muscle weakness

1	2	3	4	5
○	○	○	○	○

Physical capacity

1	2	3	4	5
○	○	○	○	○

Chronic pain

1	2	3	4	5
○	○	○	○	○

Muscle contractures

1 2 3 4 5

○ ○ ○ ○ ○

Swallowing difficulties

1 2 3 4 5

○ ○ ○ ○ ○

Malnutrition

1 2 3 4 5

○ ○ ○ ○ ○

Sexual dysfunction

1 2 3 4 5

○ ○ ○ ○ ○

Return to work

1 2 3 4 5

○ ○ ○ ○ ○

Financial stress

1 2 3 4 5

○ ○ ○ ○ ○

2.3. Rate the importance of each of the outcomes for the quality of life of FAMILIES.

Consider your experience and professional opinion. Please indicate the option that is considered most appropriate on a scale of 1 (not important) to 5 (very important).

Anxiety

1 2 3 4 5

○ ○ ○ ○ ○

Depression

1 2 3 4 5

○ ○ ○ ○ ○

Post-traumatic stress

1 2 3 4 5

○ ○ ○ ○ ○

Pathological grief

1 2 3 4 5

○ ○ ○ ○ ○

Financial stress

1 2 3 4 5

○ ○ ○ ○ ○

3. Practice

3.1. Considering PATIENTS. In the institution where you work, what priority is given to the prevention of each of the following outcomes?

Please indicate the option that is considered most appropriate on a scale of 1 (no priority) to 5 (high priority).

ICU readmission

1 2 3 4 5

○ ○ ○ ○ ○

In-hospital survival

1 2 3 4 5

○ ○ ○ ○ ○

Unplanned readmission

1 2 3 4 5

○ ○ ○ ○ ○

Survival after hospital discharge

1 2 3 4 5

○ ○ ○ ○ ○

Anxiety

1 2 3 4 5

○ ○ ○ ○ ○

Depression

1 2 3 4 5

○ ○ ○ ○ ○

Post-traumatic stress

1 2 3 4 5

○ ○ ○ ○ ○

Sleep disorder

1 2 3 4 5

○ ○ ○ ○ ○

Cognitive dysfunction

1 2 3 4 5

○ ○ ○ ○ ○

Muscle weakness

1 2 3 4 5

○ ○ ○ ○ ○

Physical capacity

1 2 3 4 5

○ ○ ○ ○ ○

Chronic pain

1 2 3 4 5

○ ○ ○ ○ ○

Muscle contractures

1 2 3 4 5

○ ○ ○ ○ ○

Swallowing difficulties

1 2 3 4 5

○ ○ ○ ○ ○

Malnutrition

1 2 3 4 5

○ ○ ○ ○ ○

Sexual dysfunction

1 2 3 4 5

○ ○ ○ ○ ○

Return to work

1 2 3 4 5

○ ○ ○ ○ ○

Financial stress

1 2 3 4 5

○ ○ ○ ○ ○

3.2. Considering FAMILIES. In the institution where you work, what priority is given to preventing each of the following outcomes?
Please indicate the option that is considered most appropriate on a scale of 1 (no priority) to 5 (high priority).

Anxiety

1 2 3 4 5

○ ○ ○ ○ ○

Depression

1 2 3 4 5

○ ○ ○ ○ ○

Post-traumatic stress

1 2 3 4 5

○ ○ ○ ○ ○

Pathological grief

1 2 3 4 5

○ ○ ○ ○ ○

Financial stress

1 2 3 4 5

○ ○ ○ ○ ○

3.3. Prevention of PICS: Which of the following strategies have been adopted in your ICU to avert undesirable post-ICU outcomes?

- ☐ An analgesia protocol
- ☐ The systematic application of pain assessment tools
- ☐ The administration of preemptive analgesia (before dressings or procedures)
- ☐ A sedation protocol
- ☐ The systematic application of sedation assessment tools
- ☐ The systematic application of delirium screening tools
- ☐ The systematic application of non-pharmacological measures to prevent delirium
- ☐ The promotion of early mobilization
- ☐ The promotion of patient sleep quality
- ☐ A flexible family visit policy
- ☐ A psychological support for patients
- ☐ A psychological support for family members
- ☐ The systematic screening of high-risk patients for post-ICU sequelae
- ☐ The systematic screening of high-risk family members for post-ICU sequelae
- ☐ A cognitive assessment before ICU discharge
- ☐ A motor assessment before ICU discharge
- ☐ A psychological assessment before ICU discharge
- ☐ None of the above

4. ICU post-discharge care plan

4.1. Does your institution have a transition process from the ICU to the ward?

- ○ Yes
- ○ No

4.2. If so, where does this follow-up take place?

- ○ Ward
- ○ After discharge
- ○ Both

4.3. Follow-up in the ward

In the event that there is no follow-up, the response should be "not applicable."

4.3.1. How many days after discharge from the ICU is the first visit? ______________________

4.3.2. Is the follow-up interdisciplinary? If so, please indicate the specialties involved.

____

____

4.3.3. What is the primary focus of the follow-up?

- ☐ Prevention
- ☐ Reabilitation
- ☐ Screening of high-risk individuals
- ☐ Research
- ☐ Other
- ☐ Not applicable

4.4. Does the institution where you work have a protocol for assessing the needs and defining the level of complexity of care that the patient will require after discharge from hospital?

- ○ Yes
- ○ No

4.5. Does the ICU team participate in the hospital discharge process?"

- ○ Only those who have been stratified
- ○ Of all patients who have passed through the ICU
- ○ No involvement

4.5.1. If the ICU team is involved in hospital discharge, who are the professionals involved?

- ☐ Physician
- ☐ Registered nurse
- ☐ Physiotherapist
- ☐ Psychologist
- ☐ Nutritionist
- ☐ Speech therapist
- ☐ Dentist
- ☐ Pharmacist

- ☐ Social worker
- ☐ Other
- ☐ Not applicable

4.6. Outpatient follow-up In the event that there is no follow-up, the response should be "not applicable."

4.6.1. How many days after discharge from the ICU is the first visit? ______________________

4.6.2. Is the follow-up interdisciplinary? If so, please indicate the specialties involved. ______________________

____

4.6.3. What is the focus of the follow-up?

- ☐ Prevention
- ☐ Reabilitation
- ☐ Screening of high-risk individuals
- ☐ Research
- ☐ Other
- ☐ Not applicable

Thank you very much!!!

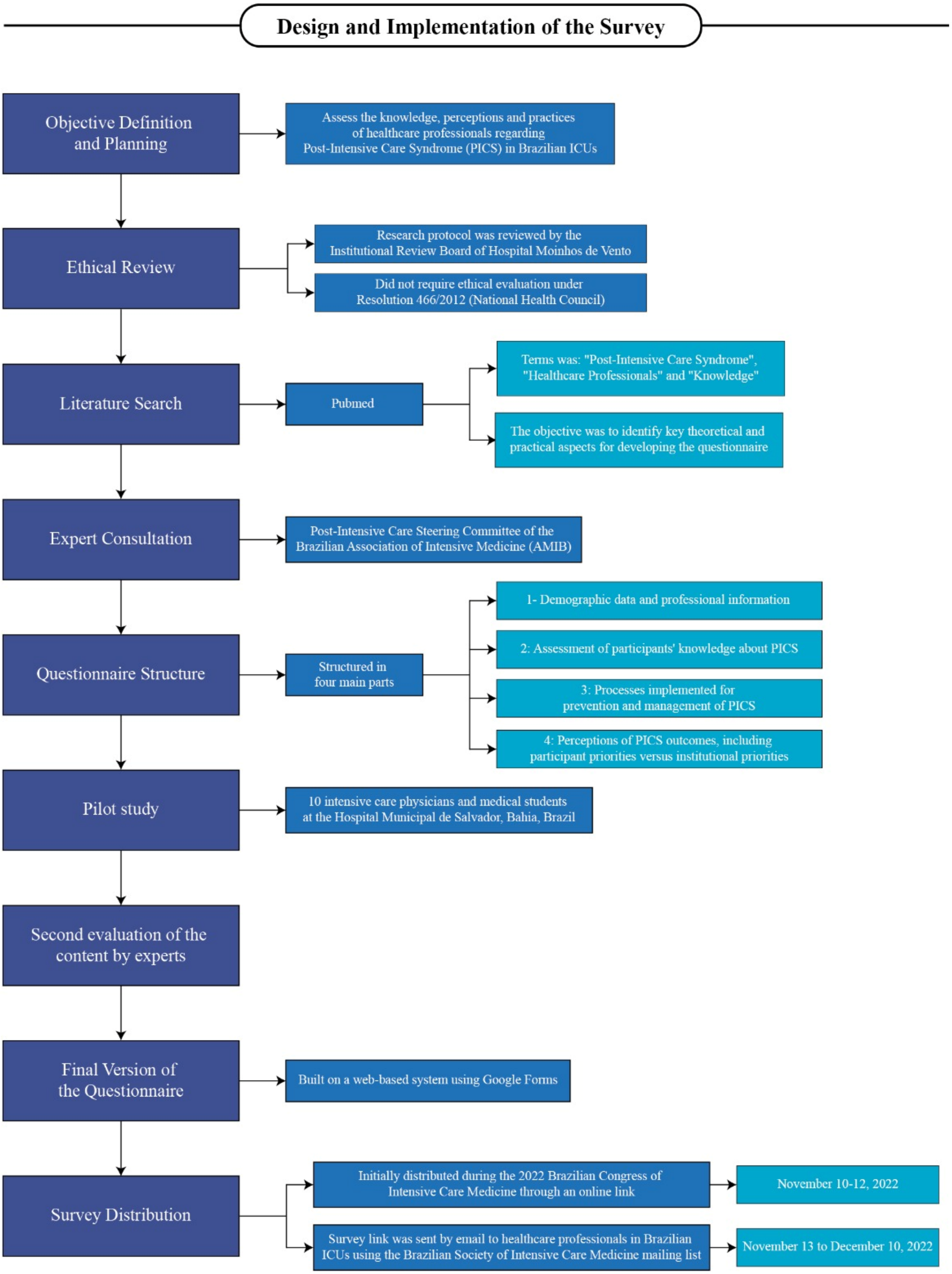

Figure 1S - Flowchart of the design and distribution of the survey.

Table 1S - Perceptions of health workers regarding post-intensive care syndrome

Perception of respondents	Value (n = 1,527)
Have you heard of post-intensive care syndrome?	1,160/1,527 (75.9)
Does your institution have a protocol for assessing the needs and determining the level of care a patient will require after hospital discharge?	403/1,527 (26.4)
Does the ICU team participate in the discharge process for patients?	
For all patients who have been through the ICU	336/1,527 (22.0)
Only those who have been stratified	274/1,527 (17.9)
Does not participate	917/1,527 (60.0)
If the ICU team participates in the discharge process, which professionals are involved?	
Social worker	202/609 (33.2)
Nurse	460/609 (75.5)
Pharmacist	115/609 (18.9)
Physiotherapist	439/609 (72.1)
Speech therapist	34/609 (5.6)
Physician	513/609 (84.2)
Nutritionist	328/609 (53.8)
Psychologist	302/609 (49.6)
Nursing technician	6/609 (0.99)
Occupational therapist	8/609 (1.3)
All health professionals	1/609 (0.16)

ICU - intensive care unit. Results expressed as n (%).
